# Anatomical Tables; Containing Concise Descriptions of the Muscles, Ligaments, Fasciæ, Blood-Vessels, and Nerves. Intended for the Use of Students

**Published:** 1840-04

**Authors:** 


ART. VIII.*—1. *Anatomical Tables, containing Concise Descriptions of the Muscles, Ligaments, Fasciæ, Blood-vessels, and Nerves. Intended for the Use of Students.* By THOMAS NUNNELEY, Lecturer on Anatomy, &c., in the Leeds School, &c.—*London*, 1838. 12mo, pp. 240.

2. *Text-Book of Anatomy for Junior Students.* By ALEXANDER J. LIZARS, M.D. Part I.—*Edinburgh*, 1839. 12mo, pp. 272.

3. *The Anatomist's Manual; or, a Treatise on the Manner of Preparing all the Parts of Anatomy, followed by a Complete Description of these Parts.* By J. P. MAYGRIER, M.D.P., &c. &c. Translated from the last French Edition.—*London*, 1839. 12mo, pp. 564.

WE have classed the above "Manuals" together, because their professed object is one and the same: viz., to assist the learner in finding the high-road to anatomical knowledge, and because neither our space nor inclination would dispose us to yield to each a separate and lengthened notice.

1. Mr. Nunneley's "Tables" are designed, as he informs us in his preface, "to assist in the understanding and recollection of anatomy," rather than to teach it; and is by no means his wish or intention that it should, in the least, usurp the place of those more extended and valuable books which are usually in the possession of the anatomical student. About this our author need not be uneasy, i. e., unless the student who employs his tables be disposed to *book* his anatomy, like a parrot. We can have no objection to Mr. Nunneley's classifying system, provided its use be limited to its intention, as a means of rubbing off the rust which will collect upon the anatomical knowledge of those who have ceased to use the knife.

2. Dr. Lizars's "Text-book" (which we presume will be completed in the second part) is a more extended manual of anatomy, well adapted for the use of the student in the dissecting-room, and, we may add, both nicely arranged and carefully and clearly written. The author's attention, in planning the compilation of a work like the present, was, as his preface informs us, drawn to the subject by "observing how much students are generally occupied, during lecture time, in taking notes of the anatomical demonstrations which are then being delivered." We quite coincide with Dr. Lizars in deprecating this practice, and have always impressed on our own pupils the folly of wasting that time in writing which should be occupied in observing, whilst the subject is purely demonstrative. Where physiology is combined, we are disposed, on the contrary, rather to encourage note-taking, within a limited compass, as the attention is thus kept alive, and the impressions so received and preserved are valuable, as being readily revived, and as forming links which are capable of recalling much more than is actually written.

3. M. Maygrier's work possesses many of the valuable characteristics of the French school of anatomy; yet we see nothing in it to make us yield the preference we have long entertained for the precise style and accurate information of Cloquet. Our author certainly begins, *ab initio*, with the "definition, origin, progress, mode of examining, and teaching anatomy;" and then proceeds to its "general and particular divisions." If we may judge by the style, the translation is accurate, even to a fault; for in almost every page the eye lights upon quaint expressions and gallicisms, which, not to mention their involving bad grammar, are disagreeable to read, and not well fitted for the use of young people. With the exception of this fault, we have no reason to complain of want of accuracy in the anatomical descriptions.

